# Formation Mechanism and Prevention of Cu Undercut Defects in the Photoresist Stripping Process of MoNb/Cu Stacked Electrodes

**DOI:** 10.3390/ma17205008

**Published:** 2024-10-13

**Authors:** Dan Liu, Liang Fang, Zhonghao Huang, Haibo Ruan, Wenxiang Chen, Jing Xiang, Fang Wu, Gaobin Liu

**Affiliations:** 1 Chongqing Key Laboratory of Interface Physics in Energy Conversion, College of Physics, Chongqing University, Chongqing 400044, China; ld54400398@163.com (D.L.); otforcwx@outlook.com (W.C.); wufang@cqu.edu.cn (F.W.); gbl@cqu.edu.cn (G.L.); 2 Chongqing BOE Optoelectronics Technology Co., Ltd., Chongqing 400714, China; huangzhonghao@boe.com.cn; 3 College of Materials Science and Engineering, Chongqing University of Arts and Sciences, Chongqing 402160, China; rhbcqu@aliyun.com (H.R.); 20190017@cqwu.edu.cn (J.X.)

**Keywords:** Cu stacked electrodes, wet strip, Cu undercut, galvanic effect, sacrificial anode, thin-film transistors (TFTs)

## Abstract

The Cu undercut is a recently discovered new defect generated in the wet stripping process of MoNb/Cu gate stacked electrodes for thin-film transistors (TFTs). The formation mechanism and preventive strategy of this defect were identified and investigated in this paper. The impact of stripper concentration and stripping times on the morphology and the corrosion potential (E_corr_) of Cu and MoNb were studied. It is observed that the undercut is Cu tip-deficient, not the theoretical MoNb indentation, and the undercut becomes severer with the increase in stripping times. The in-depth mechanism analysis revealed that the abnormal Cu undercut was not ascribed to the galvanic corrosion between MoNb and Cu but to the local crevice corrosion caused by the corrosive medium intruding along the MoNb/Cu interface. Based on this newly found knowledge, three possible prevention schemes (MoNiTi (abbreviated as Mo technology development (MTD) layer/Cu), MoNb/Cu/MTD, and MoNb/Cu/MoNb) were proposed. The experimental validation shows that the Cu undercut can only be completely eliminated in the MoNb/Cu/MTD triple-stacked structure with the top MTD layer as a sacrificial anode. This work provides an effective and economical method to avoid the Cu undercut defect. The obtained results can help ensure TFT yield and improve the performance of TFT devices.

## 1. Introduction

The thin-film transistor (TFT) is the core component of a thin-film transistor liquid crystal display (TFT-LCD) and is generally composed of a gate, a gate insulator (GI), an active layer, and a source–sdrain (S/D) electrode. For protection, each TFT device is covered with a passivation layer (PVX) [1,2]. With the development of TFT-LCDs to large sizes and high frequencies, to avoid signal delay, the conventional Al electrode material is gradually being replaced by Cu, which has better conductivity [3,4,5,6,7]. However, the adhesion strength of Cu is low, and it is easy for the film layer to peel off when Cu is directly deposited on the glass substrate as a gate. Therefore, it is necessary to first deposit the buffer layer and subsequently deposit the Cu film layer, i.e., to form a stacked electrode structure of adhesive buffer layer/Cu [8,9,10] (in this paper, the bottom layer is expressed in the front and the top one in the back). When Cu is used as the source–drain electrode, a diffusion barrier layer between the active layer and the source–drain electrode is required to prevent the Cu from diffusing into the active layer and deteriorating the performance of the TFT [11,12,13,14]. Consequently, the source–drain electrode becomes a stacked structure of the diffusion barrier layer/Cu. For Cu electrodes, there are common adhesion enhancement buffer layers and diffusion barrier layers such as Mo, Ti, MoTi, MoAl, and Cu alloys [12,15,16,17,18,19]. To suppress Cu diffusion from the top of the electrode to the GI layer or oxidation, an additional layer of protective metal can be deposited on top of the Cu layer to form a triple-stacked structure (diffusion barrier layer/Cu/protective layer or adhesion-enhancing buffer layer/Cu/protective layer) [20,21,22,23,24,25,26,27,28].

The employment of stacked structure electrodes solves the problem of the poor bonding strength of gate Cu with glass substrates and prevents the S/D Cu diffusion into the semiconductor active layers from deteriorating the TFT performance. However, each coin has two sides. The stacked electrodes bring a new challenge to the etching process. The various metal layers forming the stacked structure usually have different etched rates in the same etchant, thus causing some layers to be etched slower and less but the other to be faster and more, resulting in an irregular cross-section, uneven profile slope, or etching defects [29,30,31,32,33,34], among which the most typical one is the undercut defect; that is, the underlying metal disappears and some voids, micro-pores, or microholes occur at the bottom due to it being etched too much.

The undercut defect of the gate electrode can bring three hazards [35,36,37]: Firstly, the presence of micro-pores or -holes will affect the resistance of the electrode; secondly, the water used for cleaning in the subsequent process will remain in the micro-pores or microcracks, causing further corrosion of the Cu electrode; thirdly, it may lead to discontinuity of the GI layer, resulting in microcracks in the GI layer, along which the corrosive medium will diffuse to the gate in the follow-up process, inducing to the gate being corroded additionally. All these three influences will deteriorate the quality and yield of the devices. Therefore, it is essential to study the formation mechanism and find a way to take precautions against the undercut defect of the gate electrode.

Some works about the undercut defects in Mo/Cu stacked electrodes have been reported. For example, Yue Wu et al. found that the Cu and Mo layers in Mo/Cu stacked electrodes were slightly separated after being etched in H_2_O_2_-based etchant, and a void was formed between the Cu and Mo film, and the bottom Mo was missing; i.e., Mo undercut defects were observed [35]. Apart from the undercut phenomenon during wet etching, this defect may also occur in the stage of the photoresist (PR) stripping process. A Mo undercut was also found in Mo/Cu stacked electrodes during the PR wet stripping process by M. Chen et al. [36]. But neither of the abovementioned works proposed solutions to guard against the Mo undercut in the Mo/Cu stacked structure [35,36]. In another study, L. Guo et al. suggest that the undercut in the Mo/Cu stacked electrode was attributed to the stress difference between Mo and Cu [37], so by adjusting the deposition parameters, they changed the stress state of Mo layer from conventional compressive to tensile, which is identical to that of Cu; thus, the undercut defect was suppressed but not completely eliminated [37].

It is reported that the addition of Nb to Mo can refine the grains, increase grain boundaries, and enhance the ability to block Cu diffusion [38]. Therefore, in recent years, the MoNb/Cu stacked layer has been gradually replacing Mo/Cu as a common electrode structure in the TFT industry. In the 8.5 generation TFT mass production line, MoNb/Cu is used to fabricate the gate electrode, and the process procedures are sputtering, photolithography, wet etching, and wet stripping in sequence. After wet stripping, the manufacture of the gate electrode is finished. The next step is to deposit the GI layer by plasma-enhanced chemical vapor deposition (PECVD). At this time, the glass with the MoNb/Cu film on its top surface is used as the substrate, which usually needs to wait for some time before it enters the PECVD chamber. The waiting time in a queue in front of the PECVD machine is called Q-time (queue time) in the TFT industry. If the Q-time is too long, particles in the environment accumulate on the substrate. To avoid losing yields due to particle accumulation, the samples with long Q-time must re-experience wet stripping to be cleaned. However, in actual mass production conditions, when the samples experience more wet stripping, the undercut gradually occurs for the MoNb/Cu gate, which is manifested as the bottom MoNb being intact, but the upper Cu is separated from the MoNb, and a gap forms, and the tip of the Cu electrode is missing. Unfortunately, there are few reports about the Cu undercut of MoNb/Cu stacked electrodes in the stripping process, and solutions for this defect are lacking. Therefore, it is necessary to identify the Cu undercut formation mechanism in the wet stripping process and to propose a solution to eliminate this defect.

Thus, in this work, the undercut of a MoNb/Cu stacked gate electrode was investigated in a PR wet stripping process based on the TFT 8.5 generation production line. The cross-sectional morphology of MoNb/Cu stacked structures was microscopically observed after different stripping times, and the E_corr_ was determined by measuring the polarization curves of Cu and MoNb in the stripper solution with different concentrations. With the above analysis, the formation procedure and mechanism of Cu undercut in MoNb/Cu stacked electrodes were clarified. Then, three possible preventive structures, a Mo technology development layer (MTD, MoNiTi alloys)/Cu, MoNb/Cu/MTD, and MoNb/Cu/MoNb, were proposed, and the verification experiments were carried out. Finally, the MoNb/Cu/MTD structure was proven to be effective in completely eliminating the undercut. Our strategy to inhibit the Cu undercut defect has two advantages: One is low cost. Neither Ni nor Ti are rare metals, so the MTD alloy is cheaper than the MoNb alloy. The MoNb/Cu/MTD will not increase too much cost. The other is convenient to prepare and compatible with existing processes on the production line. To fabricate the MoNb/Cu/MTD triple-stacked structure, on the basis of the original process of MoNb/Cu structure, only one additional process of depositing MTD thin film has been added, making it easy to adjust the process and mass produce. Therefore, this work provides an effective and economical measure and guidance to remove the Cu undercut defect, which is conducive to increasing the product property and guaranteeing the TFT output under mass production conditions.

## 2. Materials and Methods

### 2.1. Preparation of Electrode Samples with Different Stacked Structures

**Figure 1:**
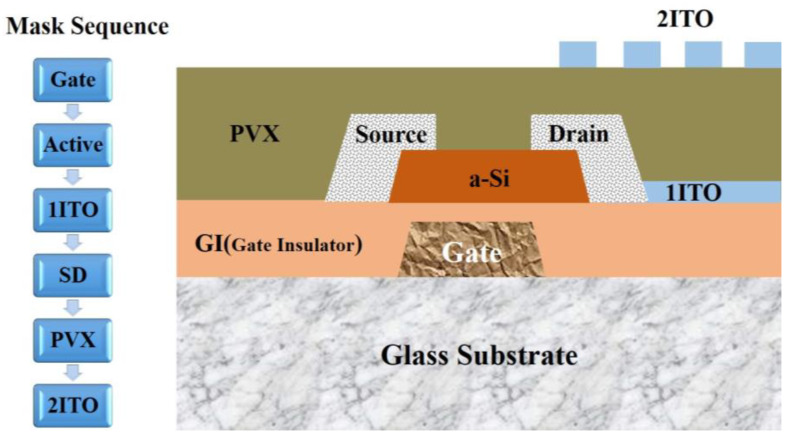
ADS Pro TFT device structure.

**Figure 2:**
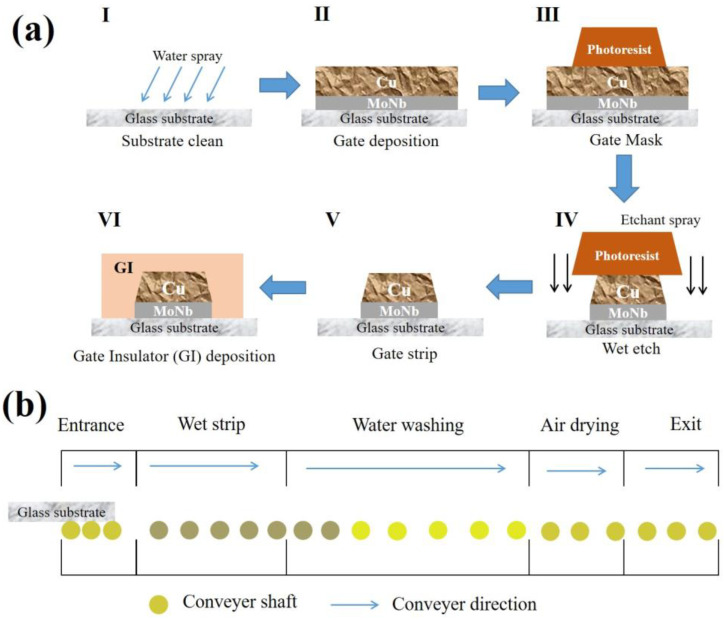
Schematic diagram of (**a**) fabrication process of gate; (**b**) wet stripping process flow.

Electrode samples with different stacked layer structures for TFT devices were prepared on the 8.5 generation TFT production line of Chongqing BOE Optoelectronics Technology Co Ltd. Figure 1 shows the TFT structure, where a gate, GI, an a-Si active layer, 1ITO, an SD electrode, a PVX layer, and 2ITO were sequentially prepared on a glass substrate to form a device. The drain electrode of the TFT was connected with the 1ITO, through which the electrical signals of the device were transmitted. The 2ITO was used as a common electrode to provide a reference voltage. The potential difference between 1ITO and 2ITO was driven to deflect the liquid crystal. The gate electrode fabrication process is displayed in Figure 2a, where the glass substrate was sequentially subjected to cleaning (Initial Clean, DMS, Yongin, Republic of Korea), deposition (2400D, ULVAC, Chigasaki, Japan), photolithography (H763, Canon, Tokyo, Japan), etching (Wet Etch, DMS, Yongin, Republic of Korea), and wet stripping (Wet Strip, DMS, Yongin, Republic of Korea) to fabricate the patterned electrodes. Then, GI deposition was performed. Figure 2b exhibits the wet stripping process, where the etched sample entered via the entrance, sequentially passed through the chambers of wet stripping, water washing, and air drying, and left the equipment via the exit. The stripping chamber and the washing chamber were, respectively, sprayed with a stripper and clean water, and the air-drying chamber was purged with CDA gas.

Three single-layer films (30 nm MoNb, 300 nm Cu, and 30 nm MTD (MoNiTi alloy)) were deposited on glass substrates by sputtering. Two double-stacked structured films (MoNb/Cu, 30/300 nm and MTD/Cu, 30/300 nm) and two triple-stacked structured electrodes (MoNb/Cu/MTD, 30/300/30 nm and MoNb/Cu/MoNb, 30/300/30 nm) were also prepared by sputtering. These stacked layer structure films were sequentially photolithographed and wet etched (H_2_O_2_ etchant, DCE-50, Dongjin Semichem Co., Ltd., Seoul, Republic of Korea) to form the electrode patterns.

### 2.2. Characterization of Films and Electrode Samples

To investigate the effect of different stripping times on Cu undercut of the stacked-structure electrodes, the cross-sectional morphology of two double-stacked-structure electrodes (MoNb/Cu, MTD/Cu) and two triple-stacked-structure electrodes (MoNb/Cu/MTD, MoNb/MoNb) were observed after etching and after stripping 1–3 times using SEM (QQSEM-01, Hitachi, Japan). The surface morphology and the surface roughness of MoNb and MTD were measured using AFM (MFP-3D-BIO, Oxford Instruments, Concord, MA, USA). For the three single-layer films of MoNb, Cu, and MTD, their contact angles (CAs) with three liquids with different polarities, water (H_2_O, polar), ethylene glycol ((CH_2_OH)_2_, polar), diiodomethane (CH_2_I_2_, nonpolar), were tested using a high-speed camera (Phantom v7.3 Vision Research, Wayne, NJ, USA). Scratch tests (G200, Agilent, Santa Clara, CA, USA) were performed on MoNb/Cu and MTD/Cu samples using a diamond indenter with a diameter of 4.6 μm, which was linearly loaded in a continuous flow at 20 mN/s with a maximum load of 300 mN. The scratches were subsequently subjected to SEM to determine the interfacial adhesion strength between buffer layer and Cu film layer based on the coarseness of the scratches.

### 2.3. Electrochemical Measurement of Electrode Samples

After the above tests were completed, the three single-layer samples of MoNb, Cu, and MTD were cut into 2 × 2 cm^2^ pieces for use as working electrodes of the electrochemical workstation (CHI660E, Shanghai Chenhua Instrument Co., Shanghai, China). The saturated calomel electrode and platinum sheet (2 × 2 cm^2^) were used as the reference and auxiliary electrodes, respectively. Polarization curves of MoNb, Cu, and MTD were tested at a scanning speed of 1 mV/s in the wet stripper (DPS-BOS-1000R, Dongjin Semichem Co., Ltd., Republic of Korea) with different concentrations (the stripper was diluted with deionized water to generate mixing solutions with different volume concentrations of stripper) to identify the corrosion potential (E_corr_) of the respective film and clarify the galvanic effects between MoNb/Cu and MTD/Cu. In addition, the polarization curves of Cu and MTD in H_2_O_2_ etchant were measured using Cu and MTD as the working electrodes; a saturated calomel electrode and a platinum sheet (2 × 2 cm^2^) were used as the reference and auxiliary electrodes, respectively, to clarify the respective E_corr_ of Cu and MTD in this environment. The electrochemical workstation (CHI660E) was used to measure the galvanic pairwise corrosion current (I_corr_) between MoNb and Cu by using a Cu sample as the working electrode, a saturated mercuric glycol electrode as the reference electrode, and a MoNb sample as the counter electrode, which were placed in the stripper with different concentrations at a scanning rate of 1 mV/s. In combination with electrochemical analysis, the formation mechanism of Cu undercut was proposed, and the solutions of MTD/Cu, MoNb/Cu/MTD, and MoNb/Cu/MoNb were recommended.

## 3. Results and Discussion

### 3.1. Effect of the Stripping Times on Cross-Sectional Morphology of MoNb/Cu Stacked Electrodes

**Figure 3:**
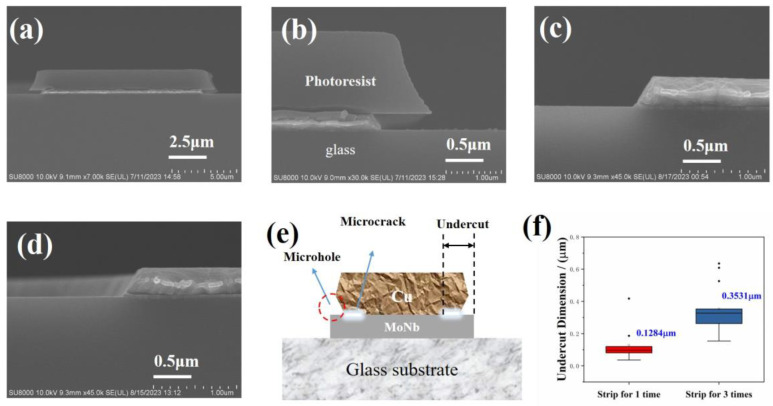
Cross-sectional morphology of MoNb/Cu after etching and stripping; (**a**,**b**) after etching; (**c**) wet stripping 1 time; (**d**) wet stripping 3 times; (**e**) schematic diagram of Cu undercut defect; (**f**) relationship between Cu undercut length and the stripping times.

The cross-sectional morphology of MoNb/Cu samples after wet etching is given in Figure 3a,b. After etching, the PR remained on the MoNb/Cu stacked structure, and a profile angle formed in the cross-section, which was smooth. The interface between MoNb and Cu was clear and complete without undercut. After the PR was stripped off, -, as shown in Figure 3c, at the position of the tip of the electrode, the bottom MoNb remained complete, but the tip of the Cu layer was missing, and there was a microcrack in the MoNb/Cu interface; i.e., Cu undercut defects occurred. The morphology of the sample subjected to three successive PR stripping is exhibited in Figure 3d, where the Cu tip missing was aggravated, and the microcrack was enlarged. From the above experiment results, it can be seen that as the wet stripping time increases, the Cu tip loss and interface microcracks become more severe. In order to quantitatively characterize the undercut, the length of the microhole and microcrack in the horizontal direction is defined as the undercut dimension by using the edge of the bottom MoNb layer as the reference point, as shown in Figure 3e. Figure 3f displays the undercut dimension after wet stripping 1–3 times for MoNb/Cu electrodes, where the Cu undercut dimension increases with more stripping time.

### 3.2. Corrosion Potentials of Cu and MoNb in the Stripper Environment with Different Concentrations

**Figure 4:**
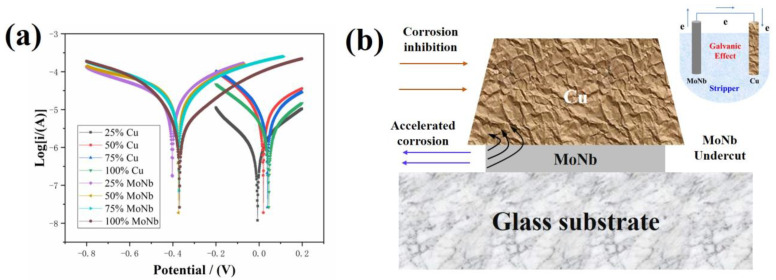
Electrochemical properties of Cu and MoNb in stripping solutions with different concentrations: (**a**) polarization curve; (**b**) schematic diagram of bottom MoNb undercut caused by MoNb/Cu galvanic effect.

The polarization curves of MoNb and Cu examined in the mixing stripper solution with 25–100 vol% concentrations are shown in Figure 4a, and the obtained E_corr_ and I_corr_ are listed in Table 1. MoNb always has a lower E_corr_ than Cu. As shown in Figure 4b, if a primary cell formed in the MoNb/Cu interface, MoNb acted as the anode due to the low E_corr_, and Cu acted as the cathode due to the high E_corr_. The anode lost electrons, and the corrosion accelerated, whereas the cathode received electrons, and the corrosion rate decreased. Therefore, MoNb will be corroded as the anode, and Cu will be protected as the cathode in the MoNb/Cu interface position at the cross-section position. At the sidewall position of the electrode, the Cu electrode sidewall area was larger than the MoNb sidewall, and a large-cathode/small-anode primary cell formed, which accelerated the anode corrosion and formed the MoNb undercut.

However, the actual undercut found here was Cu tip-deficient, not MoNb indentation, which is different from the theoretically predicted undercut defect types based on the galvanic corrosion between MoNb and Cu. Therefore, the Cu tip-deficient undercut in the MoNb/Cu stacks was not caused by the galvanic corrosion of MoNb/Cu but by some other factors.

### 3.3. Contact Angle, Surface Roughness, and Scratch Testing of Single-Layer Metal

**Figure 5:**
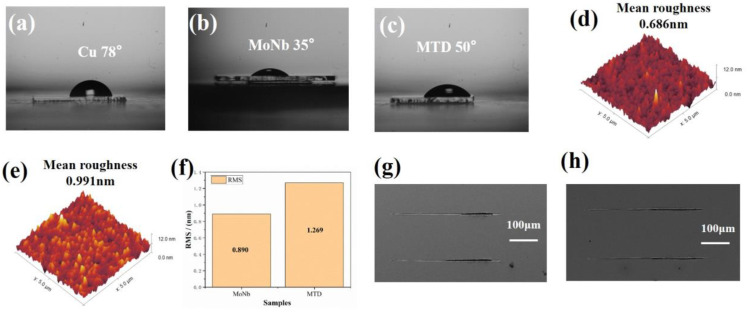
Contact angles of (**a**) Cu; (**b**) MoNb; and (**c**) MTD; surface morphologies of (**d**) MoNb and (**e**) MTD; (**f**) RMS surface roughness of MoNb and MTD; scratch morphology of (**g**) MoNb/Cu and (**h**) MTD/Cu.

The CAs of Cu, MoNb, and MTD with three liquids with different polarities, water (H_2_O), ethylene glycol ((CH_2_OH)_2_), and diiodomethane (CH_2_I_2_), were measured, among which the first two are polar, but the last one is non-polar. Figure 5a–c exhibited the CAs of Cu, MoNb, and MTD tested in water, which were 78°, 35°, and 50°, respectively. The CAs of Cu, MoNb, and MTD with the three liquids are listed in Table 2. It shows that the CAs corresponding to ethylene glycol for Cu, MoNb, and MTD are 54°, 21°, and 36°, respectively, which are in the same trend of ranking relationship of CAs as those corresponding to H_2_O. The CAs of Cu, MoNb, and MTD corresponding to diiodomethane were 37°, 33°, and 35°, respectively, with small variations in the values, but the trends of the CAs ranking relationship are still consistent with those corresponding to water.

The MoNb film layer had the smallest CA, meaning it was the most hydrophilic. And Cu had the largest CA, implying it was the most hydrophobic. The CA difference between Cu and MoNb was 33°, larger than that of Cu and MTD, 18°. M. L. Tan et al. reported that the greater the CA difference between the two materials, the lower the interfacial bonding strength [39]. So, it can be inferred that the interfacial adhesion strength between Cu and MTD is stronger than that of Cu and MoNb.

The surface morphologies of MoNb and MTD observed by AFM are demonstrated in Figure 5d,e, respectively. And the root mean square (RMS) values of surface roughness are given in Figure 5f. It shows that the mean and RMS surface roughness of MTD is 0.991 nm and 1.269 nm, respectively, larger than that of MoNb (0.686 nm and 0.890 nm), indicating that MTD has a rougher surface than MoNb. The scratch morphologies of MoNb/Cu and MTD/Cu are given in Figure 5g,h, respectively. It was obvious that the corresponding scratches of MoNb/Cu were coarser; i.e., the Cu film layer was more damaged, which indicates that the adhesion strength between Cu and MoNb is lower than that of MTD/Cu.

### 3.4. Analysis of the Formation Process and Mechanism of Cu Undercut for MoNb/Cu Stacked Electrodes

Water washing was performed immediately after stripping; thus, the stripping solution became diluted. As shown in Table 1, the E_corr_ of Cu and MoNb are gradually negatively shifted from 0.045 to −0.07 V and from −0.369 to −0.402 V, respectively, meaning both Cu and MoNb are more easily corroded in this process. But the variation in the E_corr_ negative shift of MoNb was smaller than that of Cu, indicating that the water washing process promotes Cu corrosion more than MoNb corrosion; i.e., MoNb is relatively more corrosion-resistant than Cu.

**Figure 6:**
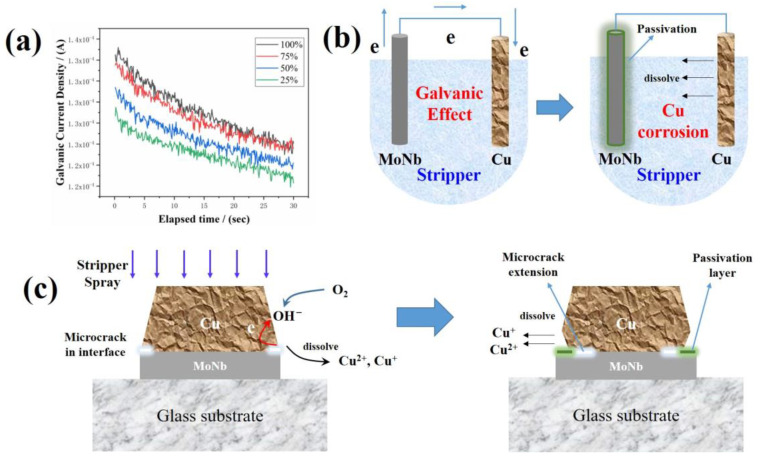
The process of MoNb/Cu galvanic corrosion and Cu undercut formation in a stripping solution environment: (**a**) variation of corrosion current density with corrosion time; (**b**) formation of the passivation layer on the MoNb surface in the MoNb/Cu primary cell; (**c**) the formation procedure of Cu undercut.

Figure 6a shows the galvanic coupling corrosion of Cu and MoNb. The pairwise corrosion current gradually decreased when the corrosion time increased. Thus, there is some substance that hinders the corrosion progress. It is reported that due to the addition of Nb, the MoNb alloy has better corrosion resistance than Mo, which is usually ascribed to a passivation layer blocking the continuous corrosion of MoNb alloy [40,41,42]. As shown in Figure 6b, the passivation layer formed on the surface of MoNb during the corrosion process, which hindered the MoNb’s direct contact with the corrosive medium. Eventually, the galvanic effect was suppressed, so the pairwise corrosion current gradually decreased. Based on this description, the Cu tip-deficient undercut formation mechanism was proposed, as shown in Figure 6c. Due to the low adhesion strength in the MoNb/Cu interface, the corrosive medium was invaded along the interface and corroded both the Cu and bottom MoNb to form microcracks. However, the MoNb surface was protected by a passivation layer to maintain a complete layer, whereas the Cu film layer could not form a passivation layer and was more susceptible to corrosion [43,44,45,46].

During the fabrication process of metal electrodes for TFT, general corrosion often hardly happens, but local corrosion may, which includes galvanic corrosion, pitting corrosion, crevice corrosion, stress corrosion, etc. In the Cu and Mo dual metal systems, galvanic and crevice corrosion were reported to be most likely to occur [36].

The typical stripper was a hydrophobic alkaline solution with organic amine which could react with copper [37]. Under alkaline electrolyte conditions, the crevice corrosion reaction of Cu involved copper dissolution and oxygen reduction as follows:

Anode: Cu → Cu^2+^ + 2e^−^; Cu → Cu^+^ + e^−^ (1)

Cathode: O_2_ + 2H_2_O + 4e^−^ → 4OH^−^ (2)

According to these two reactions, the Cu at the microcrack position withstood the continuous corrosive effect of the corrosive medium and gradually corroded and developed into a microhole. M. Chen et al. called this a crevice corrosion reaction mechanism [36], though the Cu undercut that happened in their Mo/Cu stacked structure was successfully explained.

In our case, as the stripping times increase, the degree of corrosive medium intrusion along the MoNb/Cu interface increases, which is manifested as microcrack extension. If the instances of stripping increase, the Cu corrosion at the microcrack location is increased, which is eventually manifested as Cu tip loss. In short, the Cu undercut in the MoNb/Cu stacked structure in the PR stripping process is caused by the crevice corrosion reaction.

### 3.5. New Ideas to Prevent Cu Undercut and Its Detail Design

According to the above analysis, the formation of the Cu undercut in Cu/MoNb stacked electrodes relies on two fundamental conditions: One is the microcracks, microholes, or crevice between the MoNb and Cu interface that provide the pathway for the corrosive medium; the other is the local crevice corrosion happening between the Cu in the microcracks and O_2_ in the stripper solution.

Based on this knowledge, two ideas to prevent this defect are proposed: One is to increase the adhesion strength of the buffer layer/Cu interface by replacing the MoNb alloy; the other is to change the electrode structure, such as adding a sacrificial layer to form a primary battery with O_2_ in the stripper, means, shortcut of the local crevice corrosion between the Cu and O_2_.

In the present work, MTD alloy was chosen to replace MoNb in this trial. Thus, two-layer stacked structure MTD/Cu was designed to increase the interface adhesion strength, and on the basis of MoNb/Cu, by adding a top sacrificial layer, two triple-layer stacked structures were designed, MoNb (bottom)/Cu/MoNb (top) and MoNb (bottom)/Cu/MTD (top).

So, the galvanic performance of three structures, MTD/Cu, MoNb/Cu/MoNb, and MoNb/Cu/MTD, were investigated, and the corresponding validation experiments were carried out.

### 3.6. Design of the MTD/Cu Double-Stacked Electrode and Its Effects

**Figure 7:**
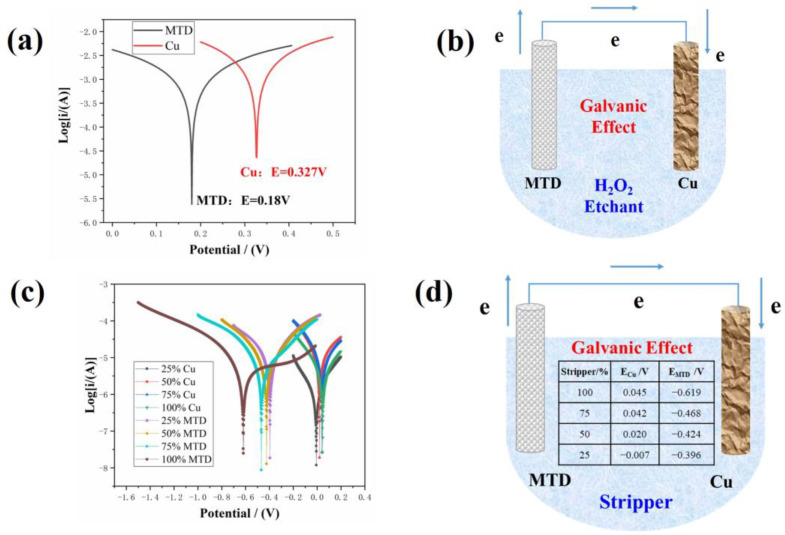
Polarization curves of Cu and MTD in the etchant (**a**) and in different concentrations of the wet stripper (**c**); schematic diagram of the MTD/Cu primary cell in the etchant (**b**) and in stripper (**d**).

The polarization curves of Cu and MTD in the H_2_O_2_-based etchant or in the mixing stripper solution with 25–100 vol% concentrations are shown in Figure 7a and Figure 7c, respectively, and the corresponding E_corr_ and I_corr_ are given in Table 1. It is seen that in the H_2_O_2_-based etching process, MTD has a lower E_corr_ than Cu, meaning that MTD and Cu formed a primary cell, in which MTD served as the anode due to its lower E_corr_. As demonstrated in Figure 7b, MTD as the anode loses electrons to accelerate the etching, and Cu as a cathode receives electrons, so the etching of Cu was inhibited.

Stripping and subsequent washing conditions were simulated based on different stripper concentrations. From Table 1, it is obvious that in the all-stripper mixture solution, MTD always has a lower E_corr_ than the Cu electrode at the same stripper concentrations, indicating the primary cell formed by MTD and Cu in the PR stripping process is similar to that in the etching process; i.e., MTD played the part of the anode to lose electrons, and Cu acted as the cathode to gain electrons. The primary cell constituted in the MTD/Cu structure in the stripping and washing chamber is demonstrated in Figure 7d. However, as the stripper concentration gradually decreases, the corresponding E_corr_ of MTD gradually increases, indicating that the corrosion becomes progressively more difficult when the stripper gets more dilute.

**Figure 8:**
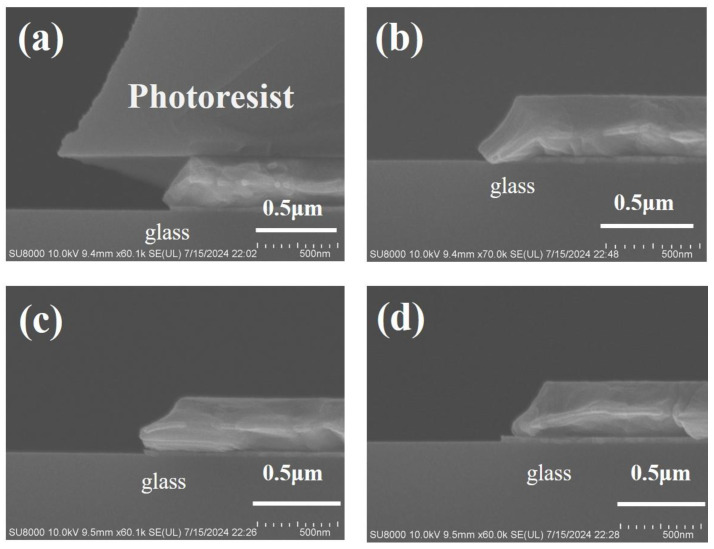
Cross-section morphology of MTD/Cu stacked structure after etching and stripping; (**a**) after etching; (**b**) stripping 1 time; (**c,d**) stripping 3 times.

The cross-sectional morphology of the MTD/Cu sample after wet etching and several instances of stripping is displayed in Figure 8. Figure 8a shows the etching defects that the bottom MTD indented inward to form the MTD undercut and the missing Cu tip in the MTD/Cu stacked structure without interface separation. The morphology of the MTD/Cu sample that underwent one time of wet stripping is exhibited in Figure 8b. It is seen that the PR was removed, and the bottom MTD undercut and the Cu tip loss were very visible.

The morphology of the sample that experienced wet stripping three times is illustrated in Figure 8c,d. Figure 8c shows the bottom MTD undercut and Cu tip missing, while Figure 8d mainly displays the interface separation between Cu and MTD and the MTD undercut. It is noted that the interface separation was not observed after one time but detected after three instances of stripping in the MTD/Cu stacked electrode. However, by contrast, the interface separation occurred in the first instance of stripping of the MoNb/Cu stacked electrode. Thus, the MTD/Cu stack has a superior inhibitory effect on interface separation to the MoNb/Cu stack.

**Figure 9:**
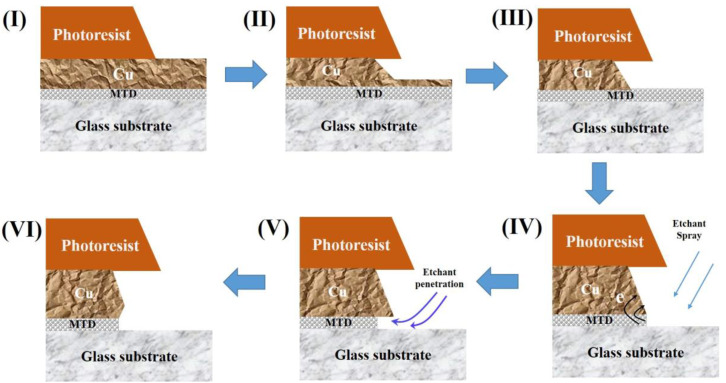
The formation process of MTD undercut in the etching process.

The formation process of the MTD undercut in the MTD/Cu structure in the etching process is illustrated in Figure 9. During the etching progressed to stage (IV), both Cu and MTD side walls were exposed to the etching solution. According to the electrochemical results of MTD/Cu given in Figure 7a and Table 1, Cu and MTD sidewalls constituted a primary cell in the etchant solution, in which the MTD served as an anode to lose electrons and accelerate the etching, and Cu acted as a cathode to accept the electrons and inhibited the etching.

Due to the larger area of the Cu sidewall, the primary cell formed between Cu and MTD is a large-cathode (Cu)/small-anode (MTD) one, in which the bottom MTD, as the small-anode, has an accelerated etching rate. Thus, at stage (V) in Figure 9, the bottom MTD undercut began to occur, and a gap was generated between the Cu layer and the glass substrate. With the etchant further intruding into the gap to etch Cu at the bottom tip position, the Cu tip was missing, and the bottom MTD undercut came into being eventually at the (VI) stage of the etching.

In summary, the MTD/Cu stacked structure has stronger interface adhesion than that of MoNb/Cu, enhancing the ability to block interface separation, but introduces the bottom MTD undercut defect in the etching process, indicating that the MTD/Cu scheme cannot complete the task to clear up the undercut defect completely.

In summary, the MTD/Cu method does not prevent the Cu undercut defect but introduces the etching defect of the bottom MTD undercut, meaning MTD/Cu did not complete the task to prevent the undercut defect.

### 3.7. Morphology of Trilayer Stacked Structure after Different Stripping Times

**Figure 10:**
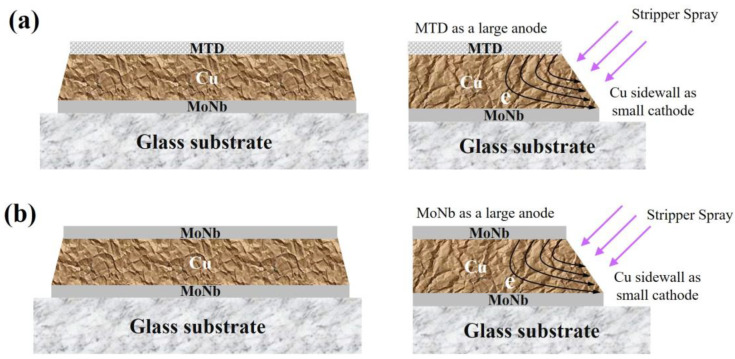
Schematic diagram of two three-layer electrode structures and their sacrificial anode schemes. (**a**) MoNb/Cu/MTD; (**b**) MoNb/Cu/MoNb.

In order to be suitable for the current mass production conditions as much as possible, the adjustment of the electrode should be in accordance with the principle of changes as small as possible. So, based on the existing MoNb/Cu stack structure, only a layer of MTD or MoNb was added to the top of the MoNb/Cu stack to form a MoNb/Cu/MTD, MoNb/Cu/MoNb triple-stacked structure, as shown in Figure 10.

As shown in Figure 10a, the PR was removed from the MTD during the PR stripping process, and a large area of the MTD was exposed to the stripper or mixture of the stripper and water. A primary cell formed between the MTD and Cu sidewall, where the MTD acted as the anode, and Cu acted as the cathode. The MTD had a large area with a micrometer width. The length of the Cu sidewall was in the nanometer scale. Thus, a large-anode (MTD)/small-cathode (Cu sidewall) primary cell was formed, the electrons in the MTD were transferred to the Cu sidewall, the concentration of electrons at the Cu sidewall location increased, and corrosion at the tip of the Cu electrode was suppressed. Although the interfacial bonding strength of MoNb/Cu was low, the interface was prone to microcrack formation, the large-anode (MTD)/small-cathode (Cu sidewall) primary cell suppressed the Cu sidewall corrosion, and microcracks at the MoNb/Cu interface could not be extended. Finally, the Cu undercut was eliminated. This method is the MTD sacrificial anode scheme. Similarly, in Figure 10b, the large area of MoNb at the top and sidewall Cu formed a primary cell, and the top MoNb acted as a sacrificial anode to transfer electrons to the Cu sidewall, which ultimately suppressed the Cu sidewall corrosion and prevented the Cu undercut.

**Figure 11:**
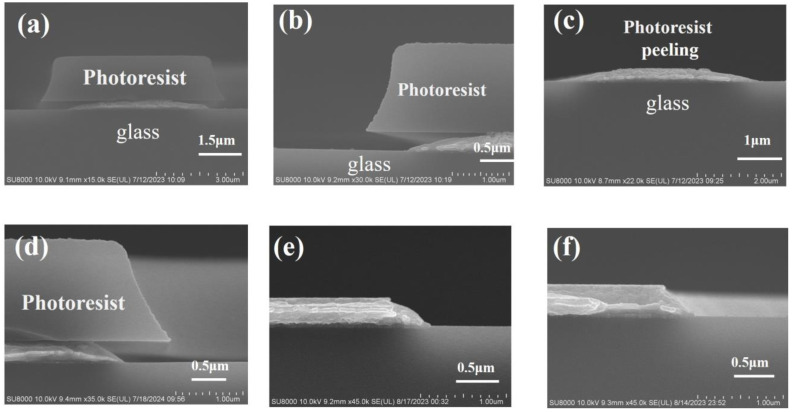
Cross-sectional morphology of two kinds of triple-layered electrodes after etching and stripping. (**a**–**c**) MoNb/Cu/MoNb after etching; (**d**) MoNb/Cu/MTD after etching, (**e**,**f**) MoNb/Cu/MTD after stripping 1 and 3 times, respectively.

Figure 11a–c shows the cross-sectional morphology of MoNb/Cu/MoNb samples after photolithography and wet etching. In Figure 11a,b, the etchant was severely laterally etched, the electrode profile angle was small, and the electrode cross-sectional area was small, which caused an abnormal increase in electrode resistance. As shown in Figure 11c, the PR at the top was peeling off during the etching process, which resulted in ineffective control of the electrode size. As shown in Figure 5a–c, the MoNb contact angle was only 35°, which was lower than that of MTD and Cu, the adhesion between MoNb and PR was the lowest, and the etchant easily penetrated from the top MoNb/PR interface, so the PR peeled off [39].

The MoNb/Cu/MTD samples were subjected to wet etching and 1–3 instances of wet stripping, and their cross-sectional morphologies are demonstrated in Figure 11d–f, respectively. Figure 11d shows the cross-section morphology after wet etching, which was smooth without undercut. Figure 11e,f shows the cross-sectional morphology of the samples after one instance and three instances of wet stripping, respectively. There was no Cu undercut in any sample, and the MoNb/Cu/MTD stacking scheme was confirmed to be effective in eliminating the Cu undercut.

To sum up, based on the understanding of the formation mechanism of the abnormal Cu undercut, which is ascribed to the local crevice corrosion caused by the corrosive medium intruding along the MoNb/Cu interface, a one bilayer staked structure (MTD/Cu) to increase the interface adhesion strength and two triple-layer stacked structures, MoNb/Cu/MoNb and MoNb/Cu/MTD, to shortcut the local crevice corrosion between the Cu and O_2_ in the stripper by adding a top sacrificial layer were designed. The experimental validation reveals that only the MoNb/Cu/MTD triple-stacked structure with a sacrificial anode of the top MTD layer can completely prevent the Cu undercut defect. There may be better choices of Mo-based alloies and optimal combinations of stacked electrode structure available in the future, but so far, the MoNb/Cu/MTD triple-stacked structure proposed in this article is the most effective and economical method to inhibit Cu undercutting.

## 4. Conclusions

In this paper, the Cu undercut defect in the TFT gate stripping process was investigated, the formation mechanism of the defect was analyzed, the solution was provided, and the effectiveness of the solution was confirmed. The main conclusions were as follows.

1. Cu undercut defects were observed in the PR wet stripping process of the MoNb/Cu stack electrode. The defects were primarily characterized by a complete MoNb bottom and a missing Cu tip with some microcracks and separation at the MoNb/Cu interface. The Cu undercut becomes severer with the increase in stripping times.
2. In the alkaline and corrosive stripper solution, MoNb has a lower E_corr_ than Cu; theoretically, a MoNb-deficient undercut should occur, but a Cu tip-deficient undercut is actually observed. So, the abnormal Cu undercut phenomenon is not ascribed to the galvanic corrosion between MoNb and Cu but to the local crevice corrosion.
3. Based on the idea of increasing the adhesion strength of the Mo-based buffer layer/Cu interface, the MTD/Cu stacked structure is proposed. It is found that due to its stronger interfacial adhesion than that of MoNb/Cu stacks, the MTD/Cu has a better ability to block interface separation and the formation of Cu undercut defects, but the Cu undercut cannot be completely eliminated in the stripping process. Moreover, MTD undercuts occurred in the wet etching process. The MTD/Cu scheme was ineffective in preventing a Cu undercut.
4. Based on the thought of adding a sacrificial anode top layer, two triple-stack structures (MoNb/Cu/MoNb and MoNb/Cu/MTD) were designed. It is verified that in the MoNb/Cu/MoNb structure, the top MoNb could be used as a sacrificial anode to suppress Cu corrosion in the stripping process. However, the PR was observed to peel off in the wet etching process, so the MoNb/Cu/MoNb stacked electrode was also invalid for removing the Cu undercut.
5. In the MoNb/Cu/MTD trilayer structure, MTD acted as a sacrificial anode, which could completely eliminate Cu undercut defects without introducing any other defects. The MoNb/Cu/MTD structure is found to be an effective scheme to inhibit Cu undercuts.
6. This work provides a strategy to avoid Cu undercut defects, which then finally contributes to improving product performance and ensuring TFT yield under mass production conditions.

## Figures and Tables

**Table 1:** Corrosion potential and corrosion current of Cu, MoNb, and MTD in stripping solution with different concentrations.

Volume Concentration	Cu		MoNb		MTD		MoNb-Cu	MTD-Cu
	E_corr_ (V)	I_corr_ (mA)	E_corr_ (V)	I_corr_ (mA)	E_corr_ (V)	I_corr_ (mA)	ΔE_corr_ (V)	ΔE_corr_ (V)
100%	0.045	2.53	−0.369	2.85	−0.619	1.93	−0.414	−0.664
75%	0.042	6.02	−0.372	9.58	−0.468	3.02	−0.414	−0.510
50%	0.020	6.81	−0.374	9.23	−0.424	3.28	−0.394	−0.444
25%	−0.007	1.06	−0.402	11.60	−0.396	4.79	−0.395	−0.389

**Table 2:** Contact angles of Cu, MoNb, and MTD with water, ethylene glycol, and diiodomethane.

	H_2_O	(CH_2_OH)_2_	CH_2_I_2_
Cu	78°	54°	37°
MoNb	35°	21°	33°
MTD	50°	36°	35°

## Data Availability

The original contributions presented in the study are included in the article, further inquiries can be directed to the corresponding author.

## References

[1] Chen W.X., Luo X., Liu D., Ling F.L., Wu F., Liu G.B., Zhang S.F., Zhang H., Li W.J., Fang L. (2024). Mechanism of electrical performance deterioration in a-Si:H TFTs caused bysource/drain Decap treatment. Microelectron. Reliab..

[2] Liu D., Huang Z.H., Wu X., Luo X., Chen W.X., Wu F., Zhang S.F., Liu G.B., Fang L. (2023). Suppression of Ion Drop of ITO-Decapped a:Si-H TFT for ADS Pro Display. SID Symaposium Dig. Tech. Pap..

[3] Arai T., Makita A., Hiromasu Y., Takatsuji H. (2001). Mo-capped Al–Nd alloy for both gate and data bus lines of liquid crystal displays. Thin Solid Film..

[4] Choe H.H., Kim S.G. (2004). Effects of the n+ etching process in TFT-LCD fabrication for Mo/Al/Mo data lines. Semicond. Sci. Technol..

[5] Nathan A., Murthy R.V.R., Park B., Chamberlain S.G. (2000). High performance a-Si H thin film transistors based on aluminum gate metallization. Microelectron. Reliab..

[6] Murarka S.P., Hymes S.W. (1995). Copper metallization for ULSL and beyond. Crit. Rev. Solid State Mater. Sci..

[7] Ho C.Y., Chang Y.J. (2016). Effects of various gate materials on electrical degradation of a-Si:H TFT in industrial display application. Solid-State Electron..

[8] Lee S.W., Cho K.S., Choo B.K., Jang J. (2002). Copper gate hydrogenated amorphous silicon TFT with thin buffer layers. IEEE Electron Device Lett..

[9] Lee S.H., Park I.S., Seo B.H., Seo J.H., Choe H., Jeon J.H., Lee Y.U., Hong M. (2011). Effect of sputtering parameters on the adhesion force of copper/molybdenum metal on polymer substrate. Curr. Appl. Phys..

[10] Wang M.C., Chang T.C., Liu P.T., Li Y.Y., Xiao R.W., Lin L.F., Chen J.R. (2007). Cu/CuMg Gate Electrode for the Application of Hydrogenated Amorphous Silicon Thin-Film Transistors. Electrochem. Solid-State Lett..

[11] Asanuma H., Suzuki T., Kusunoki T. (2012). Study on Ozonated Solution Oxidation of Phosphorus Doped Hydrogenated Amorphous Silicon Surface for Cu-Mn Alloy Based Electrodes in Thin Film Transistor. Jpn. J. Appl. Phys..

[12] Yen Y.W., Kuo Y.L., Chen J.Y., Lee C.Y., Lee C.Y. (2007). Investigation of thermal stability of Mo thin-films as the buffer layer and various Cu metallization as interconnection materials for thin film transistor–liquid crystal display applications. Thin Solid Film..

[13] Tai Y.H., Chiu H.L., Chou L.S. (2012). The deterioration of a-IGZO TFTs owing to the copper diffusion after the process of the source drain metal formation. J. Electrochem. Soc..

[14] Yim J.R., Jung S.Y., Yeon H.W., Kwon J.Y., Lee Y.J., Joo Y.C. (2012). Effects of Metal Electrode on the Electrical Performance of Amorphous In–Ga–Zn–O Thin Film Transistor. Jpn. J. Appl. Phys..

[15] Kim L.Y., Kwon O.K. (2018). Effects of Stacked Mo–Ti Cu Source and Drain Electrodes on the Performance of Amorphous In–Ga–Zn-O Thin-Film Transistors. IEEE Electron Device Lett..

[16] Kim J.L., Lee C.K., Kim M.J., Lee S.H., Jeong J.K. (2021). Role of MoTi diffusion barrier in amorphous indium-gallium-zinc-oxide thin-film transistors with a copper source drain electrode. Thin Solid Film..

[17] Yu Z.N., Xue J.S., Yao Q., Hui G.B., Jiang Y.R., Xue W. (2017). Annealing-free copper source-drain electrodes based on copper–calcium diffusion barrier for amorphous silicon thin film transistor. Thin Solid Film..

[18] Yu Z.N., Xue J.S., Yao Q., Li Z.L., Hui G.B., Xue W. (2017). The properties of Cu metallization based on CuMgAl alloy buffer layer. Microelectron. Eng..

[19] Jeong W., Winkler J., Schmidt H., Lee K.H., Park S.H.K. (2021). Suppressing channel-shortening effect of self-aligned coplanar Al-doped In-Sn-Zn-O TFTs using Mo-Al alloy source/drain electrode as Cu diffusion barrier. J. Alloys Compd..

[20] Tai M.C., Wang Y.X., Chang T.C., Huang H.C., Lin C.C., Huang B.S., Chang H.Y., Huang J.W., Sze S. (2021). Gate Dielectric Breakdown in a-InGaZnO Thin Film Transistors with Cu Electrodes. IEEE Electron Device Lett..

[21] Ma Q.G., Wang H.H., Zhang S.D., Chen X., Wang T.T. (2019). Electro-static discharge protection analysis and design optimization of interlayer Cu interconnection in InGaZnO thin film transistor backplane. Acta Phys. Sin..

[22] Ma Q.G., Zhou L.F., Yu Y., Ma G.Y., Zhang S.D. (2019). Electro-static discharge failure analysis and design optimization of gate-driver on array circuit in InGaZnO thin film transistor backplane. Acta Phys. Sin..

[23] Liu X., Wang L.L., Ning C., Hu H.H., Yang W., Wang K., Yoo S.Y., Zhang S.D. (2014). Gate bias stress-induced threshold voltage shift effect of a-IGZO TFTs with Cu gate. IEEE Trans. Electron Devices.

[24] Liu D., Huang Z.H., Wu X., Li Y.Q., Yang Y.T., Ning Z.Y., Min T.Y., Gao K.K., Fang H.L., Fang L. (2024). Effect of gate materials and stack structure on threshold voltage of ADS Pro TFT. SID Symp. Dig. Tech. Pap..

[25] Li G.T., Zhu F. (2020). Contact Performance and Thermal Stability Improvement of Amorphous InGaZnO Thin-Film Transistors by Using a Buffer/Cu/Buffer Source/Drain Electrode Structure. IOP Conf. Ser. Mater. Sci. Eng..

[26] Wu Y., Yong W.N., Chen S.J., Lee C.Y., Zhou H. A Study about the Different Corrosion Behavior between Cu/Mo Bilayer and Mo/Cu/Mo Three-layer Metal Electrode. Proceedings of the IEEE International Conference on Electron Devices and Solid State Circuits.

[27] Shin D.C., Park K.S., Park B.R., Choe H.H., Jeon J.H., Lee K.W., Seo J.H. (2011). A study on the dry etching characteristics of indium gallium zinc oxide and molybdenum by the CCP-RIE system for the 4 mask process. Curr. Appl. Phys..

[28] Chung J.M., Wu F., Jeong S.W., Kim J.H., Xiang Y. (2019). Enhanced Reliability of a-IGZO TFTs with a Reduced Feature Size and a Clean Etch-Stopper Layer Structure. Nanoscale Res. Lett..

[29] Seo B.H., Lee S.H., Park I.S., Seo J.H., Choe H.H., Jeon J.H. (2011). Effect of nitric acid on wet etching behavior of Cu/Mo for TFT application. Curr. Appl. Phys..

[30] Seo B.H., Lee S.H., Park I.S., Seo J.H., Choe H.H., Jeon J.H., Hong M.P., Lee Y.U., Winkler J. (2011). Effect of acetic acid on wet patterning of copper/molybdenum thin films in phosphoric acid solution. Thin Solid Film..

[31] Seo B.H., Lee S.H., Lee I.K., Seo J.H., Jeon J.H., Choe H.H., Lee K.W., Winkler J., Reinfried N., Knabl W. (2009). A Study on the Galvanic Reaction between Cu and Mo as Well as MoW for TFT-LCDs by Using a Zero-Resistance Ammeter. SID Symp. Dig. Tech. Pap..

[32] Liu X., Wang L.L., Hu H.H., Lu X.H., Wang K., Wang G., Zhang S.D. (2015). Performance and Stability Improvements of Back-Channel-Etched Amorphous Indium–Gallium–Zinc Thin-Film-Transistors by CF4+O2 Plasma Treatment. IEEE Electron Device Lett..

[33] Kim J.H., Bae J.W., Park J.Y., Im M.S., Kim B.O., Seo J.H. (2019). Effect of Fluoride Ions on Wet Etching of Copper/ Molybdenum in Hydrogen Peroxide Solution. J. Nanosci. Nanotechnol..

[34] Kim D.E., Cho S.W., Kim S.C., Kang W.J., Cho H.K. (2017). Corrosion Behavior and Metallization of Cu-Based Electrodes Using MoNi Alloy and Multilayer Structure for Back-Channel-Etched Oxide Thin-Film Transistor Circuit Integration. IEEE Trans. Electron Devices.

[35] Wu Y., Li S., Ge S.M., Xiong Y., Jiang C.S. (2017). A Study about the Metal Corrosion and Diffusion Phenomenon in CuMo Dual Layer Metal Wires for TFT-LCDs. SID Symp. Dig. Tech. Pap..

[36] Chen M., Li G., Sun Y., Liu H.Z., Liu J., Xia H., Tan Z.W. (2019). The Crevice Corrosion of Copper in TFT-LCD Fabrication. SID Symp. Dig. Tech. Pap..

[37] Guo L., Chen M., Sun S., Sun Y., Liu H.Z., Tan Z.W., Xiao J.C., Zhou H. (2020). Influences of Mo Film Residual Stress on Cu/Mo Interface and Undercut Performance. SID Symp. Dig. Tech. Pap..

[38] Li H.G., Xu X.C., Wang P.J., Li Q.K., Zhang T., Yang K.J., Wang J., Pan K.M., Huang Z.M., He J.L. (2022). Stability, deoxidation, and sintering characteristics of activated Mo–10%Nb solid-solution powders prepared by mechanical alloying. J. Mater. Res. Technol..

[39] Tan M.L., Liu J., Hu X.B., Liu L.F., Zhang J., Zhang C.L. (2021). Synergistic Improvement of Photoresistance Adhesion for Cu Triple-Layer Structure. SID Symp. Dig. Tech. Pap..

[40] Kostenbauer H., Lorenz D., Schober M., Winkler J. Increasing the Oxidation Resistance of Molybdenum Thin Films. Proceedings of the 59th Annual Technical Conference.

[41] Ji P.F., Li B., Chen B.H., Wang F., Ma W., Zhang X.Y., Ma M.Z., Liu R.P. (2020). Effect of Nb addition on the stability and biological corrosion resistance of Ti-Zr alloy passivation films. Corros. Sci..

[42] Chelariu R., Bolat G., Izquierdo J., Mareci D., Gordin D.M., Gloriant T., Souto R.M. (2014). Metastable beta Ti-Nb-Mo alloys with improved corrosion resistance in saline solution. Electrochim. Acta.

[43] Wang H., Sun Z.M., Gao P., Tu L.Z., Mo C.D., Sun S.Y., Zhang W.M. (2019). Study of the Copper Corrosion by EUV and TMAH in Lithography Cleaning Process. SID Symp. Dig. Tech. Pap..

[44] Lin M., Liu Z.Y., Wei Y., Ma Y., Liu B., Meng Y., Qiu H. (2021). Cu Erosion of Via Hole within GOA Unit of Super-large and Ultra-HD TFTLCD of 120Hz Frame Rate. SID Symp. Dig. Tech. Pap..

[45] Ye C., Yang Y.T., Zhou L.X. (2021). Analysis and Research of Pad Corrosion of COG LCD. SID Symp. Dig. Tech. Pap..

[46] Yang L., Yu X., Zhang Z.Q., Zhao S., Zhang P.P., Song R.C. (2023). Adjusting Cu layer thickness of ITO/Cu/ITO film to improve electrochemical corrosion of GOA unit. J. Alloys Compd..

